# A replicative recombinant HPV16 E7 expression virus upregulates CD36 in C33A cells

**DOI:** 10.3389/fmicb.2023.1259510

**Published:** 2023-08-30

**Authors:** Yunting Shao, Peng Wang, Yunji Zheng, Hongtu Cui, Zhangrong Lou, Shanhu Li, Fang Huang, Chengjun Wu

**Affiliations:** ^1^School of Biomedical Engineering, Dalian University of Technology, Dalian, China; ^2^Department of Cell Engineering, Beijing Institute of Biotechnology, Beijing, China; ^3^School of Pharmacy, Binzhou Medical University, Yantai, China

**Keywords:** high-risk HPV, HPV16 E7, Ad4, recombinant HPV16 E7 expression virus, CD36

## Abstract

**Objective:**

In past decades, the role of high-risk HPV (HR-HPV) infection in cancer pathogenesis has been extensively studied. The viral E7 protein expressed in pre-malignant cells has been identified as an ideal target for immunological intervention. However, the cultivation of HPV *in vitro* remains a significant challenge, as well as the lack of methods for expressing the HPV E7 protein and generating replication-competent recombinant viral particles, which posed a major obstacle to further exploration of the function and carcinogenic mechanisms of the E7 oncoprotein. Therefore, it is imperative to investigate novel methodologies to construct replication-competent recombinant viral particles that express the HPV E7 protein to facilitate the study of its function.

**Methods:**

We initiated the construction of recombinant viral particles by utilizing the ccdB-Kan forward/reverse screening system in conjunction with the Red/ExoCET recombinant system. We followed the infection of C33A cells with the obtained recombinant virus to enable the continuous expression of HPV16 E7. Afterwards, the total RNA was extracted and performed transcriptome sequencing using RNA-Seq technology to identify differentially expressed genes associated with HPV-induced oncogenicity.

**Results:**

We successfully established replicative recombinant viral particles expressing HPV16 E7 stably and continuously. The C33A cells were infected with recombinant viral particles to achieve overexpression of the E7 protein. Subsequently, RNA-Seq analysis was conducted to assess the changes in host cell gene expression. The results revealed an upregulation of the CD36 gene, which is associated with the HPV-induced oncogenic pathways, including PI3K-Akt and p53 signaling pathway. qRT-PCR analysis further identified that the upregulation of the CD36 gene due to the expression of HPV16 E7.

**Conclusion:**

The successful expression of HPV16 E7 in cells demonstrates that the replicated recombinant virus retains the replication and infection abilities of Ad4, while also upregulating the CD36 gene involved in the PI3K-Akt signaling and p53 pathways, thereby promoting cell proliferation. The outcome of this study provides a novel perspective and serves as a solid foundation for further exploration of HPV-related carcinogenesis and the development of replicative HPV recombinant vaccines capable of inducing protective immunity against HPV.

## 1. Introduction

The human papillomavirus (HPV) is a small (8,000 bp), non-enveloped double stranded circular DNA virus (Pan et al., 2021; Sun et al., 2021; Wang et al., 2022). Persistent infection with HR-HPVs is highly related with various cancers, including cervical cancer (99.7%), head and neck squamous cell carcinomas (60%), anal cancer (93%), vulvar cancer (69%), vaginal cancer (75%), and penile cancers (47%) (Yang et al., 2019; Meznad et al., 2021). As the most prevalent HR-HPV, HPV16 accounts for approximately 50% of cervical cancer cases (Qmichou et al., 2013), its genome is composed of 6 early genes (E6, E7, E1, E2, E4, and E5) and 2 late genes (L1 and L2) (Zhu et al., 2021). Early genes are responsible for regulating viral transcription and genome replication, while late genes encode capsid proteins and glycoproteins. The sustained expression of E6 and E7 in cell lines has been demonstrated to induce immortalization and transformation in various rodent and human cells (Hawley-Nelson et al., 1989; Hoppe-Seyler et al., 2018; Lou et al., 2022). In some rare cases, long-term persistent HR-HPV infection results in the integration of the oncogenes E6 and E7 into the host DNA (Schneider et al., 2020). This integration disrupts host cell apoptosis and promotes continuous cell proliferation, ultimately leading to cancer development. E7 interacts with pRB, resulting in the inactivation of pRB function and the dissociation of E2F from the E2F/pRB complex, which triggers the G1/S transition (Sitarz et al., 2021). This disruption further enhances the proliferation and transformation of epithelial cells. HPV infection-related malignancies remain a significant global public health concern, especially in developing countries. As the most efficient method to prevent HPV infection, HPV prophylactic vaccine has been introduced for more than a decade (Zhou et al., 2019; Yang et al., 2022). However, prophylactic HPV vaccines only target the HPV late genes L1 and/or L2. However, in high-grade lesions or HPV-associated malignancies, the prophylactic vaccine is ineffective since L1 and L2 are lost due to HPV integration into the host genome (Cheng et al., 2018). Previous studies demonstrated that the consistent overexpression of HPV E7 oncoproteins is required throughout the process of cervical epithelial cell carcinogenesis (Domingos-Pereira et al., 2021). Hence, HPV E7 gene is an optimal target for the treatment and prevention of HPV-induced cancers (Domingos-Pereira et al., 2021; Peng et al., 2022). However, since HPV life cycle is tightly linked to the host cell differentiation, the virus is extremely difficult to isolate and culture (Wang et al., 2022). Therefore, the construction of recombinant viruses is of great significance in overcoming these obstacles and paving the way for further exploration of the pathogenesis and treatment of HPV-related cancers.

Adenovirus (Ad) is one of the most commonly used vectors for gene therapy and possesses many advantages over other viral vectors, including high transduction efficiency, broad tissue affinity, and non-integration into the host genome (McKenna et al., 2020; Liu et al., 2021). The utilization of Ad as a vector offers several benefits, such as the effective transduction of target cells at a low multiplicity of infection (MOI), the presence of well-established techniques for manipulation and propagation, and a relatively safe profile due to the viral genome is not integrated into the host genome. Additionally, Ad vectors can deliver large therapeutic genes (approximately 37 kb) (Douglas, 2007; Wu et al., 2015). It is worth mentioning that a previous study indicated that CR1 and CR2 in Ad E1A contain the pRb binding domain, and the Ad E1A (12S) protein shares structural and functional similarities with the HPV E7 protein (Lou et al., 2022; Nouel and White, 2022). The E7 N terminus comprises two regions that exhibit sequence similarity with a segment of conserved region 1 (CR1) and conserved region 2 (CR2) found in the adenovirus E1A protein (Ad E1A) (Nouel and White, 2022). Similar to the Ad E1A antigen, the HPV E7 proteins interact with the retinoblastoma tumor suppressor protein pRB and the related “pocket proteins” p107 and p130 through a conserved LXCXE sequence within CR2 sequences (Dürst et al., 1983; Dyson et al., 1992). These pocket proteins play a crucial role in regulating the activities of the E2F family of transcription factors, which control multiple cell cycle transitions and other cellular activities (Cam and Dynlacht, 2003). The ability of HPV E7 and Ad E1A antigen to associate with pRB plays a critical role in their ability to generate and/or maintain a host cellular milieu conducive to viral genome replication. Several studies have provided evidence that the E6 and E7 proteins of HPV play a role in supporting the DNA replication of Ad that lack the E1A and E1B genes (Steinwaerder et al., 2001), which suggests HPV E7 protein may serve as partial substitutes for Ad E1 proteins in the replication of viral DNA. Hence, we posit that the integration of HPV E7 into the Ad E1A region to generate a replicative recombinant virus capable of expressing HPV E7.

However, due to the significant disparity in size between the Ad genome (approximately 36 kilobases) and the HPV16 E7 gene (a mere 270 base pairs), the precise integration of the HPV16 E7 gene into the E1A region of Ad in a single attempt presents a big challenge. Hence, we designed a novel approach to accurately generate a replicative recombinant HPV16 E7 expression virus (Ad4-HPV16E7), which involved the integration of the HPV E7 gene into the EGFP-Tagged Ad4 E1A region utilizing the ccdB-Kan forward/reverse screening system in conjunction with the Red/ExoCET recombinant system. The establishment of this approach can provide novel ideas and basis for further investigations into the role of HPV16 E7 in cancer pathogenesis, as well as potential therapeutic approaches targeting viral infections and tumor growth.

## 2. Materials and methods

### 2.1. Bacterial strains, plasmids, cell culturing

All *Escherichia coli* strains and plasmids used in this study are listed in Supplementary Table S1. *Escherichia coli* strains were grown in LB medium at 30°C or 37°C and selected with appropriate antibiotics [chloramphenicol (Cm), 10 μg/mL; ampicillin (Amp), 10 μg/mL; kanamycin (Kan), 10 μg/mL and tetracycline (Tet), 34 μg/mL]. The concentration of 10% L-arabinose used for induction was 25 mg/mL. Ad4 was purchased from ATCC. C33A cells was purchased from Cell Resource Center of Peking Union Medical College. HEK293T, Siha, Hela and Caski cells were preserved in our laboratory. The cells were maintained in DMEM (EallBio, China) or MEM, RPMI1640 supplemented with 10% FBS and 1% penicillin–streptomycin (P/S) and incubated at 37°C with 5% humidified CO_2_.

### 2.2. Molecular docking

To simulate Ad4 E1A-HPV16 E7 highly accurate 3D homology model structures, Autodock Vina 1.2.2, a silico protein–protein docking software (Zhang et al., 2023) was employed. The 3D coordinates of Ad4 E1A (PDB ID, 2R7G; resolution, 1.67 Å) and HPV16 E7 (PDB ID, 4YOZ; resolution, 2.25 Å) were downloaded from the PDB.^1^ All protein files were converted into PDBQT format with all water molecules excluded and polar hydrogen atoms were added. Molecular docking studies were performed by Autodock Vina 1.2.2.^2^

### 2.3. Generation of recombinant virus Ad4-HPV16E7

#### 2.3.1. Construction of recombinant HPV16E7 expression virus plasmid

Combination of ccdB screening system and Red-recombination to construct recombinant HPV16E7 expression virus plasmid. Only plasmids carrying ccdB gene can survive in ccdB-resistant GB08Red gyr462 strains. CcdB and Kan genes was inserted into EGFP-Tagged Ad4 E1A region, then electroporated into *E. coli* GB08Red gyr462 electrocompetent cells for Red-recombination (Zhou et al., 2019; Huang et al., 2021; Yang et al., 2022) to construct recombinant ccdB-Kan expression vector, and positive recombinant bacteria were screened in LB solution containing kanamycin. The recombinant ccdB-Kan expression vectors were, respectively, used to construct recombinant HPV16E7 virus plasmid PBR322-Ad4-E1Amut-C16E7P by subsequent ExoCET recombination (Yu et al., 2019; Schneider et al., 2020; Sitarz et al., 2021).

#### 2.3.2. Generation of recombinant virus particles

The recombinant HPV16E7 expression virus plasmid PBR322-Ad4-E1Amut-C16E7P was linearized with *AsisI* digestion to released recombinant viral DNA Ad4-HPV16E7 and purified by Phenol-chloroform extraction followed by ethanol precipitation. Lipo-3000 (ThermoFisher, USA) was used to transfect Ad4-HPV16 E7 in HEK293T cells to produce viral particles. To verify the cervical cancer cells infectivity of the recombinant viruses, SiHa, Caski, HeLa and C33A cells were infected with recombinant virus.

#### 2.3.3. Determination of virus titer

HEK293T cells were infected with different concentrations of Ad4 and Ad4-HPV16E7 virus, and without virus infection as control group. Ten multiple Wells were set up in each group, and the fluorescence number of cells in each well was counted each 2 days until the 10th day of culture. There were 10 multiple wells in each group, and the fluorescence points of each well were counted each 2 days until the 10th day of culture. On the 10th day, the number of fluorescent spots in Ad4 and Ad4-HPV16E7 virus infected cells were counted, and then TCID50 was calculated by the Karber method.

### 2.4. qRT-PCR analysis

To assess the transcript levels of the E7 gene of the Ad4-HPV16E7 virus, C33A cells were infected with the recombinant virus Ad4-HPV16 and Ad4 (control group) for a duration of 24 h. Subsequently, the RNA of the cells was collected and subjected to qRT-PCR to determine the levels of E7 gene transcripts. Primers for qRT-PCR detection of HPV16 E7 were16E7-F (forward primer, 5-AGGAGGAGGATGAAATAGATGG-3) and 16E7-R (reverse primer, 5-GCACAACCGAAGCGTAGA-3); Primers for qRT-PCR detection of GPDH were GPDH-F (forward primer, 5- GGAAGGTGAAGGTCGGAGTC -3) and GPDH-R (reverse primer, 5- GAAGGGGTCATTGATGGCAAC -3).

### 2.5. Western blotting

To evaluate Hexon/E7 protein expression levels of Ad4-HPV16E7 virus, HEK293T cells were infected with recombinant virus Ad4-HPV16E7 and Ad4 (control group) respectively for 24 h, without viral infection were used as a blank control group, then the cell total protein was extracted with a cell lysis buffer (120 mM NaCl, 0.5% NP-40, 50 mM Tris–HCl pH 8.0, and 1 mM PMSF) and evaluated by BCA methods. Whole cell extracts (30–45 μg) were eparated by 12% SDS-PAGE and then transferred to NC membranes and incubated with 5% skim milk in TBST at room temperature for 1.5 h. After that, the membranes were probed with primary antibodies at a 1:200–1:500 dilution overnight at 4°C: Rabbit anti-HPV 16 E7 antibody (cat. no. 67017-1-Ig; ProteinTech Group, Inc., Chicago, IL, USA), mouse anti-Hexon antibody (generated in our laboratory), and mouse anti-GPDH antibody (cat. no. 4970; Cell Signaling Technology, Inc., Beverly, MA, USA), then washed with TBST and incubated with goat anti-rabbit (1:5,000; Sigma)/mouse (1:10,000; Sigma) horseradish-peroxidase conjugated secondary antibody for 1 h at room temperature for 2 h. Detection was performed by Gel Detection System (Bio-Rad, USA). Western blotting bands were quantified using ImageJ software.^3^

### 2.6. Immunofluorescence assays

To confirm the Western Blot results about E7 protein expression levels of Ad4-HPV16E7 virus, HEK293T cells were infected with recombinant virus Ad4-HPV16E7 for 24 h. After that, the cells infected with Ad4-HPV16E7 virus was washed three times with PBS for 10 min per wash, fixed in 4% paraformaldehyde, and permeabilized with 0.1% Triton X-100, then blocked with 1% BSA. HPV16 E7 in cells was detected with monoclonal rabbit anti-HPV 16 E7 antibody and a secondary goat anti-mouse Alexa Fluor 594 antibody (Invitrogen, 1:1,000) and visualized by fluorescence microscopy.

### 2.7. RNA-Seq sample collection and preparation

#### 2.7.1. RNA quantification and qualification

C33A cells were subjected to infection with recombinant virus Ad4-HPV16E7 and Ad4 (control group) for a duration of 24 h. A blank control group was also included, which did not undergo viral infection. Total RNA was extracted from all groups using Trizol Reagent (Thermo Fisher Scientific). The RNA Nano 6000 Assay Kit of the Bioanalyzer 2100 system (Agilent Technologies, CA, USA) was employed to assess the quantity and quality of RNA.

#### 2.7.2. Library preparation for transcriptome sequencing

The RNA sample preparations utilized total RNA as the input material. The mRNA was isolated from the total RNA through the utilization of poly-T oligo-attached magnetic beads. Fragmentation was achieved via divalent cations at an elevated temperature in First Strand Synthesis Reaction Buffer (5X). The first strand cDNA was synthesized utilizing random hexamer primer and M-MuLV Reverse Transcriptase, followed by RNA degradation with RNaseH. Subsequently, the second strand cDNA synthesis was performed utilizing DNA Polymerase I and dNTP. The residual overhangs were transformed into blunt ends through the utilization of exonuclease/polymerase activities. Following the adenylation of the DNA fragment’s 3′ ends, an adaptor with a hairpin loop structure was ligated to facilitate hybridization. To isolate cDNA fragments ranging from 370 to 420 base pairs in length, the library fragments underwent purification using the AMPure XP system (Beckman Coulter, Beverly, USA). Subsequently, the PCR product was purified using AMPure XP beads, and the library was ultimately obtained via PCR amplification. Following the construction of the library, it was initially assessed using the Qubit2.0 Fluorometer, subsequently diluted to 1.5 ng/μL, and the insert size was determined using the Agilent 2100 bioanalyzer. Upon meeting the expected insert size, qRT-PCR was employed to precisely quantify the effective concentration of the library, ensuring that it surpasses 2 nM and meets the requisite quality standards.

#### 2.7.3. Clustering and sequencing

Once the library has met the necessary qualifications, the various libraries are combined based on their effective concentration and target data volume from the machine, and subsequently sequenced using the Illumina NovaSeq 6000 platform, resulting in a paired-end read of 150 bp. The sequencing process follows the principle of Sequencing by Synthesis, whereby synthesis and sequencing occur simultaneously. The sequenced flowcell was subjected to the addition of four fluorescent labeled dNTP, DNA polymerase, and splice primers, followed by amplification. As the sequence cluster extends the complementary chain, the fluorescence-labeled dNTPs release corresponding fluorescence. The sequencer captures the fluorescence signal and converts it into a sequencing peak through computer software, thereby enabling the acquisition of sequence information pertaining to the fragment under examination.

### 2.8. RNA-Seq data analysis

#### 2.8.1. Data analysis quality control

The high-throughput sequencer measures image data which is subsequently converted into sequence data (reads) through CASAVA base recognition. The raw data (raw reads) in fastq format undergo initial processing via fastp software, resulting in the acquisition of clean data (clean reads) by eliminating reads containing adapter, N base, and low quality reads from the raw data. Additionally, the Q20, Q30, and GC content of the clean data are computed. The downstream analyses rely on the high-quality clean data.

#### 2.8.2. Permission to reuse and copyright

The reference genome and gene model annotation files were obtained directly from the genome website. The reference genome was indexed using Hisat2 (v2.0.5), and the paired-end clean reads were aligned to it using the same tool. The selection of Hisat2 as the mapping tool was based on its ability to generate a splice junction database from the gene model annotation file, resulting in superior mapping outcomes compared to non-splice mapping tools.

#### 2.8.3. Quantification of gene expression level

The quantification of gene expression levels was performed using featureCounts (v1.5.0-p3) to enumerate the number of reads mapped to each of the two genes. Subsequently, the Fragments Per Kilobase of transcript sequence per Millions base pairs sequenced (FPKM) was computed for each gene, taking into account both the gene length and the mapped reads count. FPKM is a widely adopted method for estimating gene expression levels, as it accounts for the impact of sequencing depth and gene length on the reads count.

#### 2.8.4. Differential expression analysis

The DESeq2 R package (version 1.20.0) was utilized to conduct differential expression analysis between two conditions/groups, each with two biological replicates. DESeq2 employs statistical routines based on the negative binomial distribution to determine differential expression in digital gene expression data. The resulting *p*-values were subjected to the Benjamini and Hochberg’s approach for controlling the false discovery rate. Significantly differential expression was defined as padj<=0.05 and|log2(foldchange)| > = 1, which served as the threshold.

#### 2.8.5. Enrichment analysis of differentially expressed genes

The Gene Ontology (GO) enrichment analysis was performed on the differentially expressed genes using the clusterProfiler R package (version 3.8.1), with correction for gene length bias. GO terms that demonstrated a corrected *p*-value of less than 0.05 were considered significantly enriched by the differentially expressed genes. The KEGG database serves as a valuable resource for comprehending the overarching functionalities and utilities of biological systems, encompassing the cell, organism, and ecosystem, through the analysis of molecular-level data, particularly extensive molecular datasets produced by genome sequencing and other advanced experimental methodologies.^4^ The statistical enrichment of differential expression genes in KEGG pathways was assessed using the clusterProfiler R package (version 3.8.1). The Reactome database, which consolidates the reactions and biological pathways of human model species, was utilized. Reactome pathways exhibiting a corrected *p*-value of less than 0.05 were deemed significantly enriched by differential expressed genes. Additionally, the DO (Disease Ontology) database, which characterizes the function of human genes and diseases, was employed. DO pathways with a corrected *p*-value of less than 0.05 were also considered significantly enriched by differential expressed genes. The integration of human disease-related genes is facilitated by the DisGeNET database. DisGeNET pathways exhibiting a corrected *p*-value of less than 0.05 were deemed significantly enriched by differential expressed genes. The statistical enrichment of differentially expressed genes in the Reactome pathway, the DO pathway, and the DisGeNET pathway was assessed using the clusterProfiler R package (version 3.8.1).

### 2.9. Protein–protein interactions network

The STRING database^5^ aims to provide a comprehensive assessment and integration of PPIs, encompassing both indirect (functional) and direct (physical) associations (Xia et al., 2020). To evaluate the interactive relationship among differentially expressed genes (DEGs), the DEGs were firstly mapped to STRING. Only experimentally validated interactions with a combined score > 0.4 were considered as significant. Subsequently, the PPIs network was constructed using the Cytoscape software (Ver. 3.7.1). The plug-in Molecular Complex Detection (MCODE) was employed to screen the modules of PPIs network in Cytoscape.

### 2.10. Statistical analysis

All the experiments were conducted for three independent repeats. Statistical analysis was conducted using GraphPad Prism software (GraphPad Software). Data are presented as the mean ± SD. Student’s t test analysis was performed in differences measured variables between experimental and control groups. One-way ANOVA was performed between multiple groups and conditions. Differences were considered significant when *p* < 0.05.

## 3. Results

### 3.1. Ad E1A and HPV E7 are capable of forming a stable protein docking model

**Figure 1:**
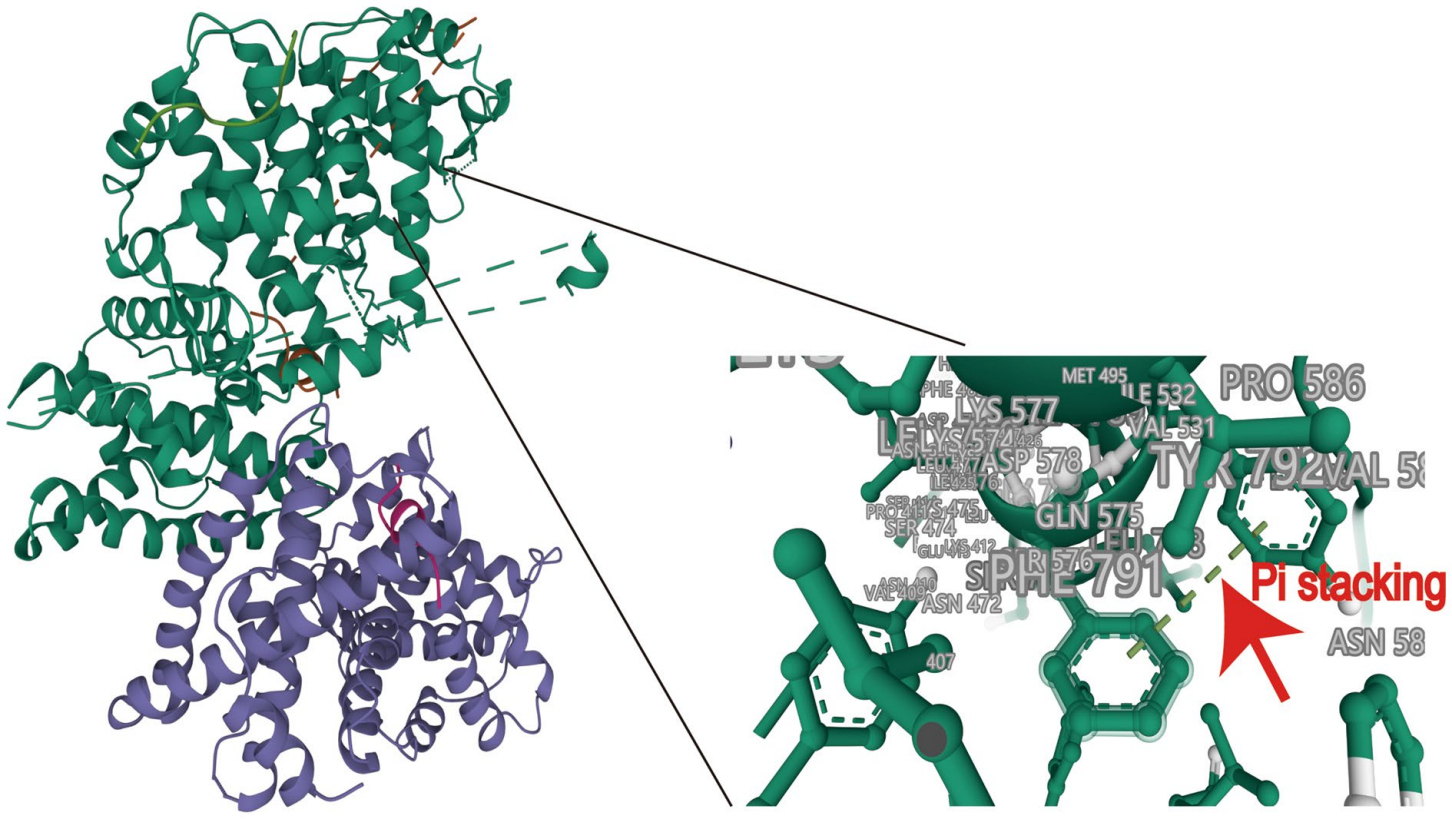
Molecular docking of HPV16 E7 onto Ad4 E1A and protein docking model. A docking score of ≥1,000 was considered acceptable.

To validate the feasibility of incorporating HPV E7 into Ad E1A, we conducted molecular docking analysis to assess their binding affinity and interaction modes. The ZDOCK score values and their Pi stacking interaction mode are shown in Table 1. The ZDOCK Score of Ad4 E1 and HPV E7 was 1202.281, and a docking score of ≥1,000 was considered acceptable. HPV E7 forms several Pi stacking interactions with amino acid sites of Ad E1A such as PHE791-TYR792, HIS554-PHE551, which was indicated by the red arrow in Figure 1. Comprehensive analysis revealed that proteins Ad4 E1A and HPV E7 formed a stable protein docking model.

**Table 1:** Results of molecular docking.

Receptor	Ligand	ZDOCK score	Pi stacking interaction
Ad E1A (2R7G)	HPV E7 (4YOZ)	1202.281	A: PHE791[occupancy 7]- A: TYR792[occupancy 3] A: HIS554[occupancy 4]- A: PHE551[occupancy 7] A: TYR756[occupancy 3]- A: TYR709[occupancy 3]

### 3.2. The recombinant virus Ad4-HPV16E7 express HPV16 E7 gene

**Figure 2:**
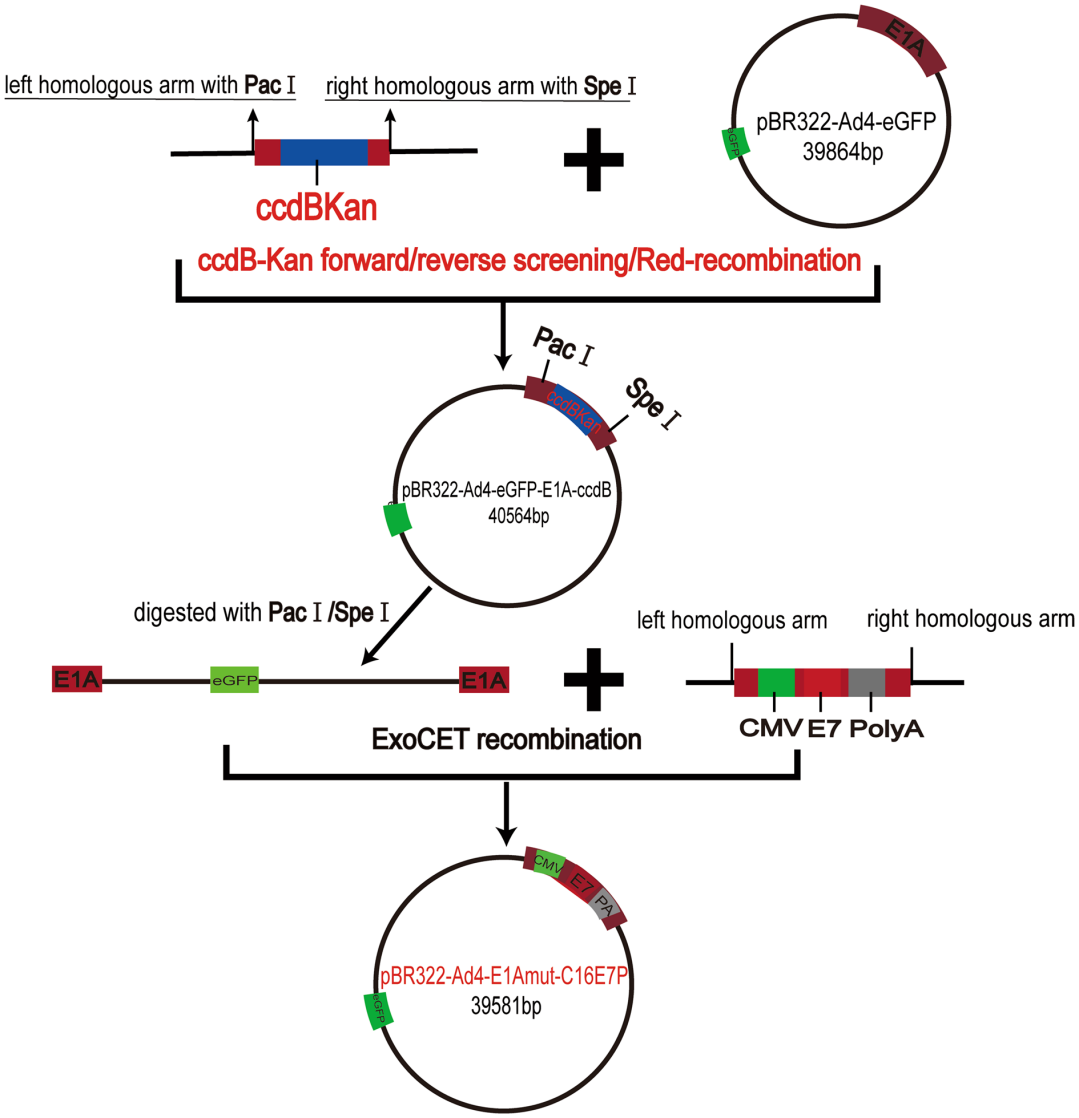
Schematic diagram of the recombinant virus plasmids PBR322-Ad4-E1A mut-C16E7P constructs.

We constructed the recombinant virus plasmids PBR322-Ad4-E1Amut-C16E7P using the ccdB-Kan forward/reverse screening system in combination with the Red/ExoCET recombinant system to precisely insert the HPV E7 gene into the EGFP-Tagged Ad4 E1A region (Figure 2).

**Figure 3:**
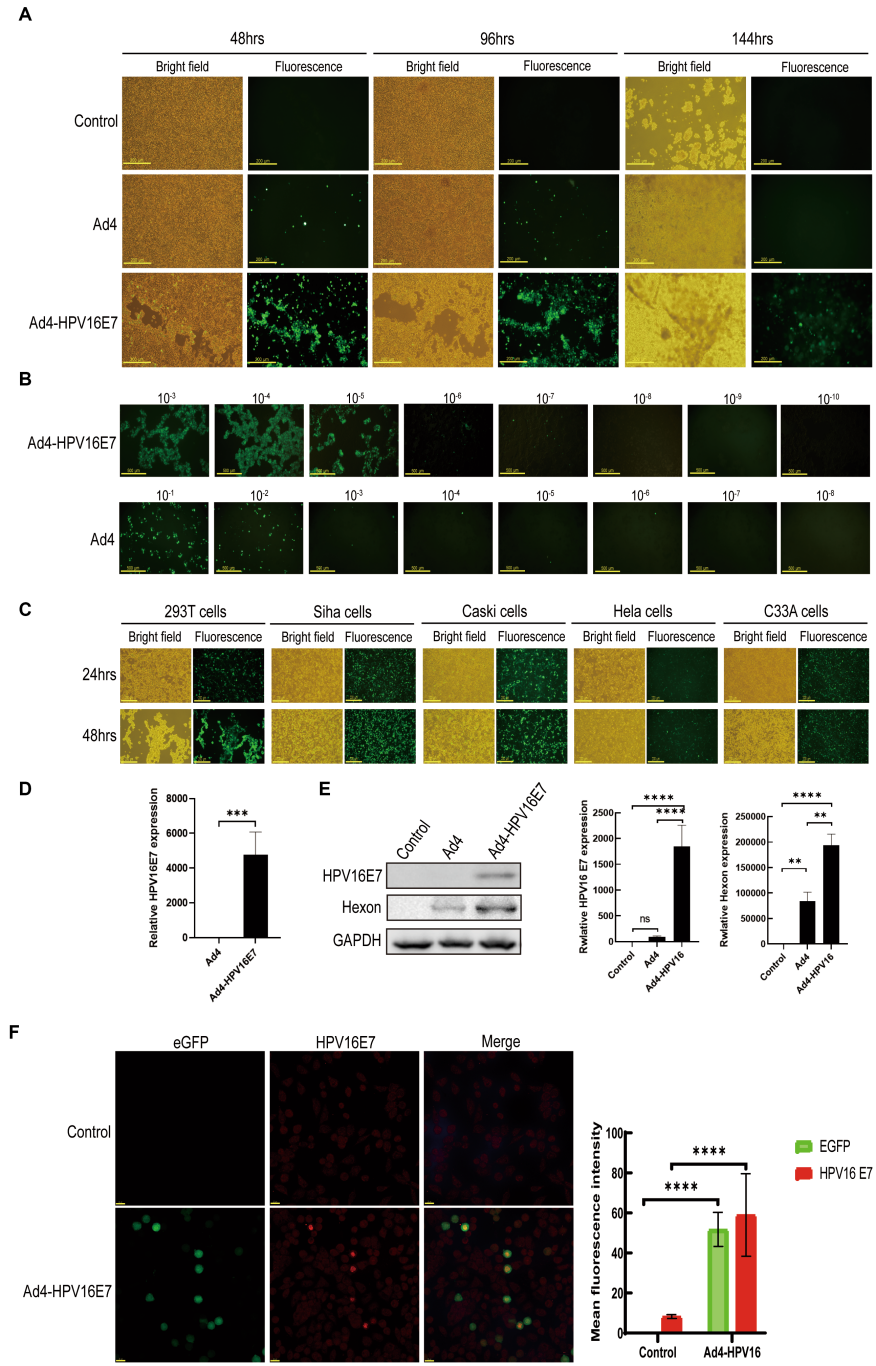
Generation of the recombinant virus Ad4-HPV16E7 and evaluation of the HPV16 E7 gene. **(A)** HEK293T cells were transfected with the pAd4-HPV16E7 and pAd4 to obtain the recombinant viruses Ad4-HPV16E7 and Ad4. **(B)** HEK293T cells were infected with eight 10-fold serial dilutions of Ad4-HPV16E7 and Ad4 viruses to calculate the viral titer. **(C)** HEK293T, SiHa, Caski, HeLa, and C33A (HPV negative) cells were able to express the GFP protein. **(D)** RT-qPCR analysis revealed that the HPV16 E7 gene was exclusively detected in C33A cells after the infection of Ad4-HPV16E7 virus. **(E)** The expression of HPV16 E7 and Hexon proteins were detected by Western Blot. **(F)** The expression of HPV16 E7 protein were detected by cellular immunofluorescence assay. Control: cells that are not infected with virus; Ad4, Cells infection with Ad4 virus; Ad4-HPV16E7, Cells infection with Ad4- HPV16E7 virus. *P < 0.05, **P < 0.01, ***P < 0.001, and ****P < 0.0001 were considered statistically significant; ns, non-significant.

To obtain the recombinant viruses Ad4-HPV16E7 and Ad4, HEK293T cells were transfected with plasmids pAd4-HPV16E7 and pAd4 for 24–144 h. Fluorescence observations indicated a significantly higher level of GFP expression in cells infected with Ad4-HPV16E7 compared to cells infected with Ad4. The optimal infection time was between 48 and 96 h (Figure 3A). At 96 h, we collected the medium containing Ad4 and recombinant virus Ad4-HPV16E7, labeling them as the P0 generation. Subsequently, we repeated the infection of HEK293T cells using the P0 medium of Ad4 and Ad4-HPV16E7 for 96 h, resulting in the generation of the P1 viral-containing medium. This procedure was repeated until we obtained the P3 generation viral medium. The viral titer of the recombinant virus Ad4-HPV16E7 in the P3 generation exhibited a substantial increase (Ad4: TCID50 = 10^–7.34^/0.1 mL, Ad4-HPV16:TCID50 = 10^–6.75^/0.1 mL) (Figure 3B). 24-h infection of HEK293T cells yielded an infection efficiency exceeding 80% by utilizing the P3 viral culture supernatant (Figure 3C), which shortened the experimental duration. Consequently, all subsequent experiments involved a 24-h infection of cells using the P3 viral medium. In order to evaluate the infectivity of the recombinant virus in different cell lines, we conducted infection experiments on SiHa (HPV16 positive), Caski (HPV16 positive), HeLa (HPV18 positive), and C33A (HPV negative) cells. The results indicated that after a 24-h infection, all four cell lines were able to express the GFP protein. However, the infection efficiency in Hela cells was significantly lower compared to SiHa, Caski, and C33A cells (Figure 3C).

We utilized the generated recombinant virus Ad4-HPV16E7 to infect the HPV-negative cell line C33A to evaluate the stability of HPV16 E7 gene transcription. RT-qPCR analysis revealed that the presence of the HPV16 E7 gene was exclusively detected in the Ad4-HPV16E7 group, while no amplification signal was observed in the Ad4 group as expected (Figure 3D). These results indicate that the HPV16 E7 gene of the recombinant virus Ad4-HPV16 was effectively transcribed in C33A cells.

Further investigation was conducted to assess the expression of HPV16 E7 protein by the recombinant virus Ad4-HPV16E7. Western blot analysis demonstrated that HEK293T cells infected with Ad4-HPV16E7 exhibited the expression of HPV16 E7 protein, whereas E7 expression was not detected in cells infected with Ad4 virus or in the control group (Figure 3E). To further identify the expression of HPV16 E7 protein by Ad4-HPV16E7, cellular immunofluorescence assay was performed, as shown in Figure 3F. The results demonstrated that the expression of HPV16 E7 protein (bright red) was observed in C33A cells infected with the recombinant virus Ad4-HPV16E7, while no such expression was observed in the control group. The primary capsid protein of adenovirus, known as Hexon (Wodrich et al., 2003), indirectly reflects the virus replication capacity through its expression level. Therefore, we also wish to verify whether the recombinant virus maintained the replication ability of Ad4. The expression of Hexon protein in HEK293T cells infected by Ad4 and Ad4-HPV16E7 was evaluated. The results revealed that both Ad4 and Ad4-HPV16E7-infected cells expressed Hexon protein. Interestingly, the expression of Hexon in the recombinant virus Ad4-HPV16E7 was higher compared to the Ad4 virus (Figure 3E), further suggests that replication capacity of Ad4-HPV16E7 might be better than Ad4 virus.

### 3.3. A total of 525 genes were up-regulated and 1029 genes were down-regulated in C33A cells infected with the Ad4-HPV16E7 virus compared to that with Ad4

**Figure 4:**
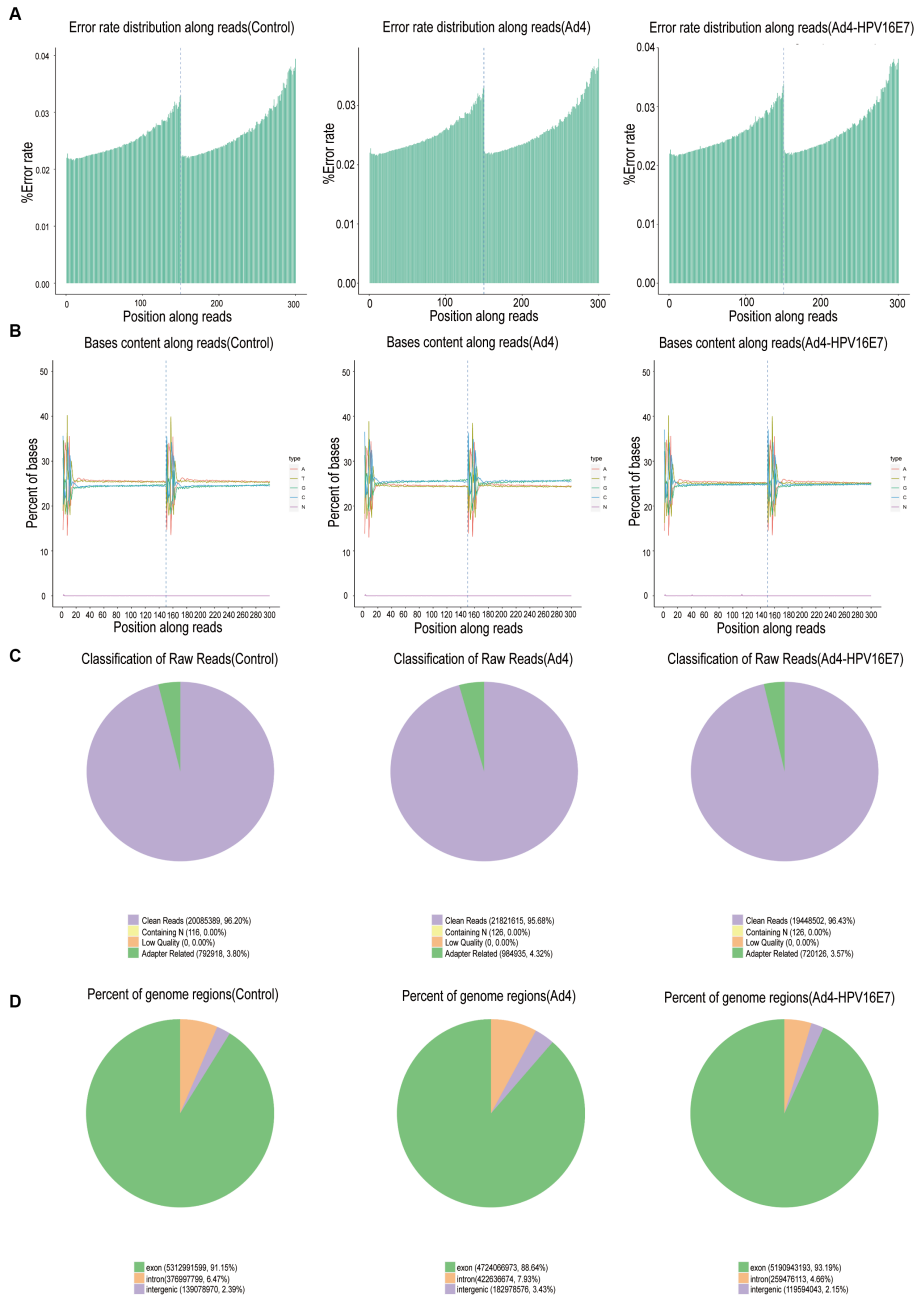
Basic information on RNA-Seq. **(A)** Distribution of sequencing data error rate. Data with an error rate below 0.02% was selected for analysis. **(B)** Distribution of GC content. **(C)** The filtered sequencing data of per sample. **(D)** Distribution of sequencing reads in different regions of the genome. The threshold range of the percentage of clean reads greater than 80% indicates that qualified for downstream analyses.

C33A cells were infected by recombinant Ad4-HPV16E7 and Ad4 viruses for 24 h. Total RNA extracted from the cells served as input for RNA sample preparations. Data with an error rate below 0.02% (Figure 4A) and stable GC content were selected for analysis (Figure 4B). Raw sequencing data underwent filtration to ensure data quality and reliability. Following filtration, the clean reads percentages were 96.20, 95.68, and 96.43% for the control, Ad4, and Ad4-HPV16E7 groups, respectively (Figure 4C). The HISAT2 software precisely aligned the clean reads with the reference genome, providing positional information (Dai et al., 2022). Read counts in exonic, intronic, and intergenic regions were determined. The proportion of clean reads in the exon region was 91.15, 88.64, and 93.19% for the control, Ad4, and Ad4-HPV16E7 groups, respectively (Figure 4D).

**Figure 5:**
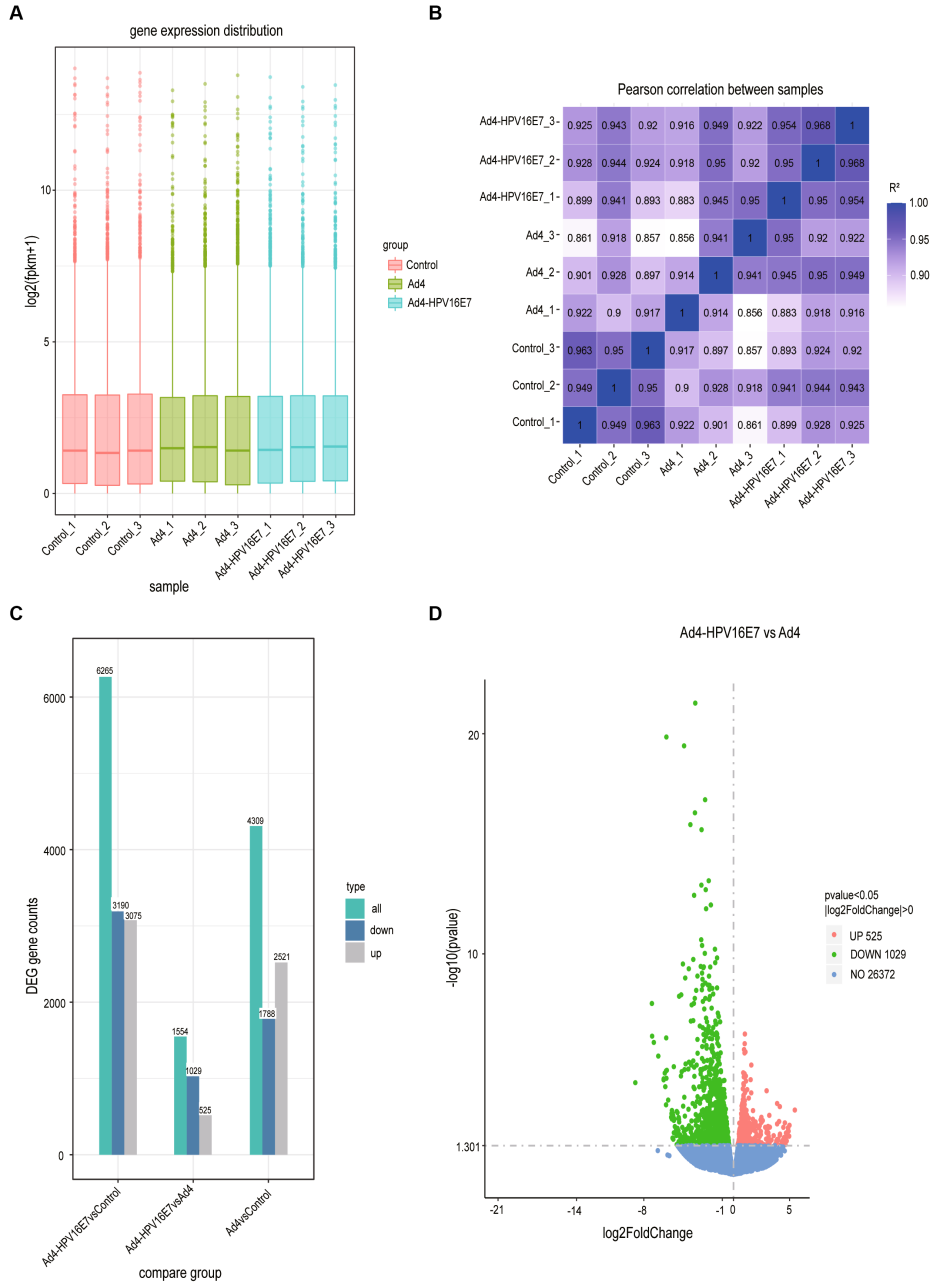
The comparative analysis of differentially expressed genes (DEGs). **(A)** Box plot of sample gene expression distribution. **(B)** Heat map of correlation between samples. *R*^2^ > 0.8 were considered a strong correlation. **(C)** Statistical histogram of the number of significantly DEGs in the different comparison combination. **(D)** Volcano map of DEGs between the Ad4-HPV16E7 group and Ad4 group.

The expression values of all genes (FPKM) in each sample were computed, and box plots were used to visualize the distribution of gene expression levels in different samples (Figure 5A). Correlation coefficients were calculated based on FPKM values to assess sample correlations within and between groups, which were further utilized to generate the sample correlation heat map (Figure 5B). Subsequently, the comparative analysis of differentially expressed genes (DEGs) was conducted. The statistical histogram revealed 4,309 DEGs in the Ad4 group compared to the control group, while the Ad4-HPV16E7 group exhibited 6,265 DEGs compared to the control group (Figure 5C). To examine the distribution of genes showing significant expression variations between the Ad4-HPV16E7 group and Ad4 group. The volcano plot (Figure 5D) illustrated 525 up-regulated genes and 1,029 down-regulated genes in the Ad4-HPV16E7 group compared to the Ad4 group.

### 3.4. A biologically interconnection of DEGs as a collective entity

**Figure 6:**
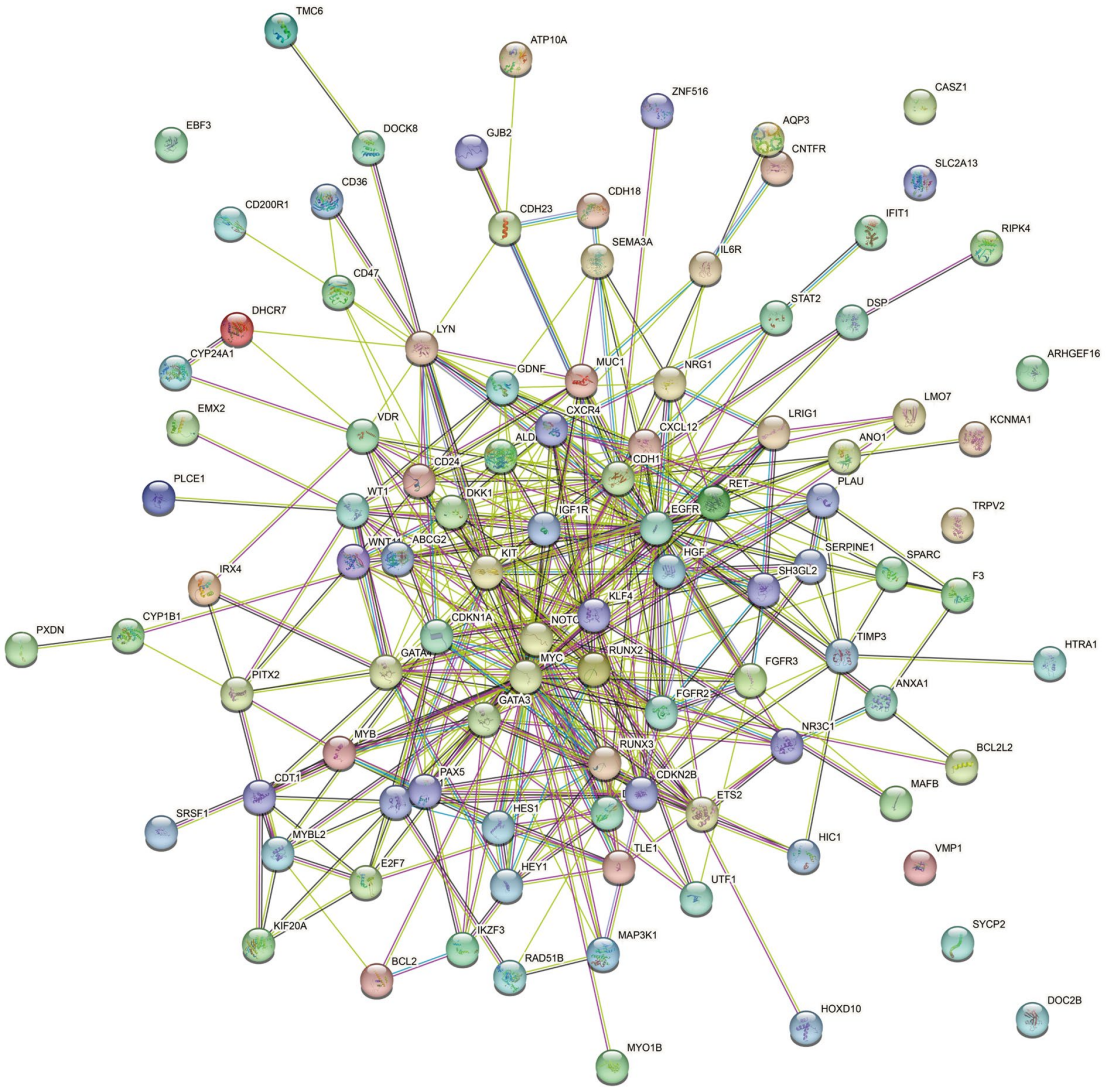
STRING protein–protein interaction of the 103 DEGs genes. Each node symbolizes the entirety of proteins generated by an individual gene locus responsible. Colored nodes are query proteins and first shell of interactors. Edges represent protein–protein associations. Associations are meant to be specific and meaningful, i.e., proteins jointly contribute to a shared function.

A comprehensive gene search was conducted using OMIM^6^ and Genotype^7^, resulting in the identification of 39 genes and 377 genes related to HPV infection, respectively. PubMed^8^ was extensively reviewed, yielding 1,172 genes associated with HPV infection, and an additional 162 genes were obtained from the human papillomavirus infection pathway (hsa05165) in KEGG. These genes were then cross-screened with significantly different genes obtained from RNA-Seq sequencing results. After filtering, a total of 103 differentially expressed genes (DEGs) were identified as HPV infection-related. The filtered genes were further analyzed using the STRING database to construct a protein–protein interactions (PPIs) network. The resulting network consisted of 100 nodes, 398 edges, and an expected number of 133 edges. The average node degree was 7.96, average local clustering coefficient was 0.566, and the PPI enrichment *p*-value was <1.0^−16^ (Figure 6). This enrichment suggests a biologically interconnected nature of the proteins within the network as a collective entity.

### 3.5. HPV-associated up-regulated genes STAT2, CD36, IFIT1, VMP1, and MUC1 of DEGs

This study conducted an analysis of the 103 DEGs genes associated with HPV infection using GO, KEGG, DO, and DisGeNET. The analysis revealed enriched biological processes, metabolic pathways, and disease associations. The results were visualized through bubble diagrams, presenting the top enriched terms and pathways.

**Figure 7:**
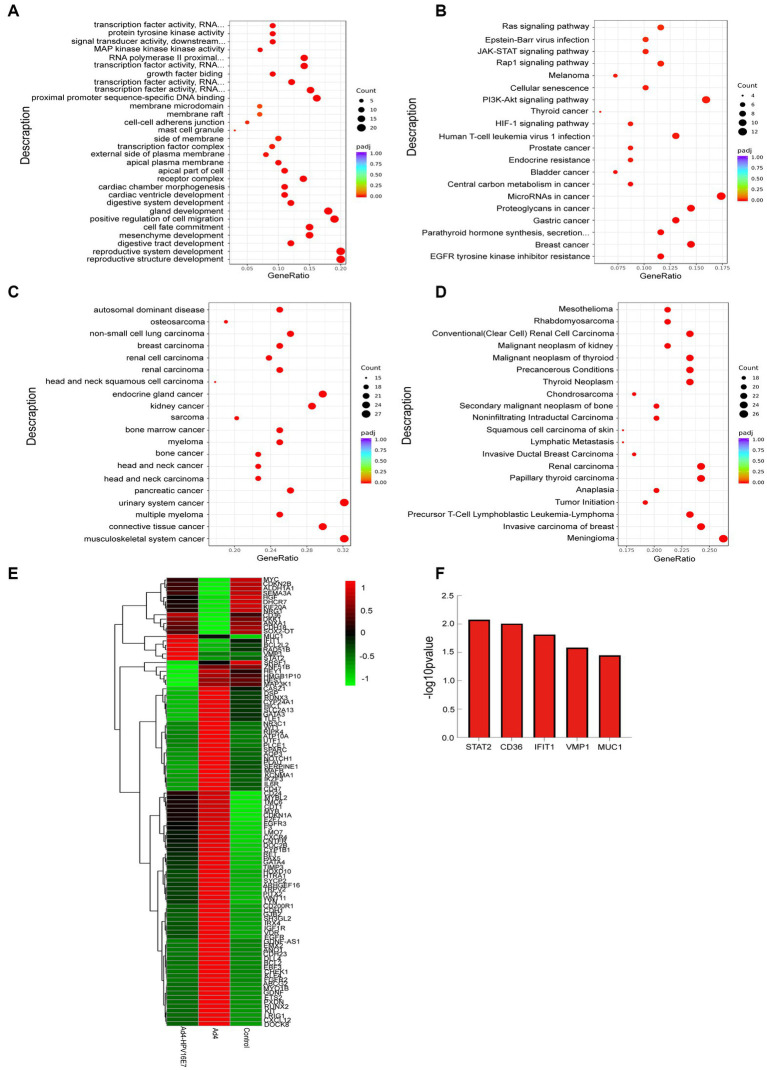
Functional and pathway enrichment analysis of identified modules associated with the 103 DEGs. **(A)** Gene Ontology (GO) functional analysis of DEGs. **(B)** KEGG pathway analysis of DEGs. **(C)** DO pathway analysis of DEGs. **(D)** DisGeNET pathway analysis of DEGs. The size of the dots represents the number of genes annotated to the KEGG pathway, and the color from red to purple represents the significance level of KEGG pathway enrichment, the redder the color is, the more significant the result. **(E)** The heat map of the expression levels of 103 genes in the Ad4-HPV16E7, Ad4, and control groups. The greener the color of the heatmap, the lower the gene expression, and the redder the color of the heatmap, the higher the gene expression. **(F)** The five up-regulated genes in the Ad4-HPV16E7 group relative to the Ad4 group and the control group.

The GO enrichment analysis of biological processes (BP) identified that the 103 genes were mainly involved in the reproductive system, positive regulation of cell migration, gland development, cell fate commitment, mesenchyme development, digestive system and cardiac ventricle development. The analysis of cellular components (CC) indicated enrichment in receptor complexes, apical regions of cells, membrane sides, transcription factor complexes, membrane microdomains, cell–cell adherens junctions, and specific granule membranes. The analysis of cellular components (CC) indicated enrichment in receptor complexes, apical regions of cells, membrane sides, transcription factor complexes, membrane microdomains, cell–cell adherens junctions, and specific granule membranes (Figure 7A). The Kyoto Encyclopedia of Genes and Genomes (KEGG) pathway analysis highlighted 103 genes were involved in MicroRNAs in cancer, PI3K-Akt signaling pathway, breast cancer, proteoglycans in cancer, human T-cell leukemia virus 1 infection, gastric cancer, EGFR tyrosine kinase inhibitor resistance, prostate cancer, thyroid cancer, Rap1 signaling pathway, JAK–STAT signaling pathway and Ras signaling pathway (Figure 7B). The DO pathway analysis indicated these genes were mainly enriched in head and neck cancer, urinary system cancer, breast carcinoma, endocrine gland cancer and musculoskeletal system cancer (Figure 7C). Furthermore, the DisGeNET pathway analysis indicated these genes were mainly enriched in meningioma, invasive carcinoma of breast, papillary thyroid carcinoma, renal carcinoma, precursor T-cell lymphoblastic leukemia-lymphoma, precancerous conditions and tumor initiation (Figure 7D). Cluster analysis was conducted on the expression levels of 103 DEGs genes in the Ad4-HPV16E7, Ad4, and control groups, as depicted in the heat map (Figure 7E). Based on cluster analysis and incorporating previously reported HPV infection-related genes from the literature, we further screened five up-regulated genes (STAT2, CD36, IFIT1, VMP1, and MUC1) in the Ad4-HPV16E7 group that were expressed relative to the Ad4 and control groups (Figure 7F).

### 3.6. Infection of recombinant virus Ad4-HPV16E7 induces upregulation of CD36 gene in C33A cells

Recent studies indicate that the presence of E7 in HPV-positive cells disrupts the tumor suppressor pRb and affects various signaling pathways. The PI3K/Akt signaling cascade is particularly significant in HPV-induced carcinogenesis. Our KEGG pathway analysis confirmed the significant involvement of the 103 genes in the PI3K/Akt signaling pathway. Previous research has highlighted the importance of the PI3K/AKT/mTOR network in mediating communication between HPV-positive cancer cells under normoxic and hypoxic conditions (Bossler et al., 2019).

**Figure 8:**
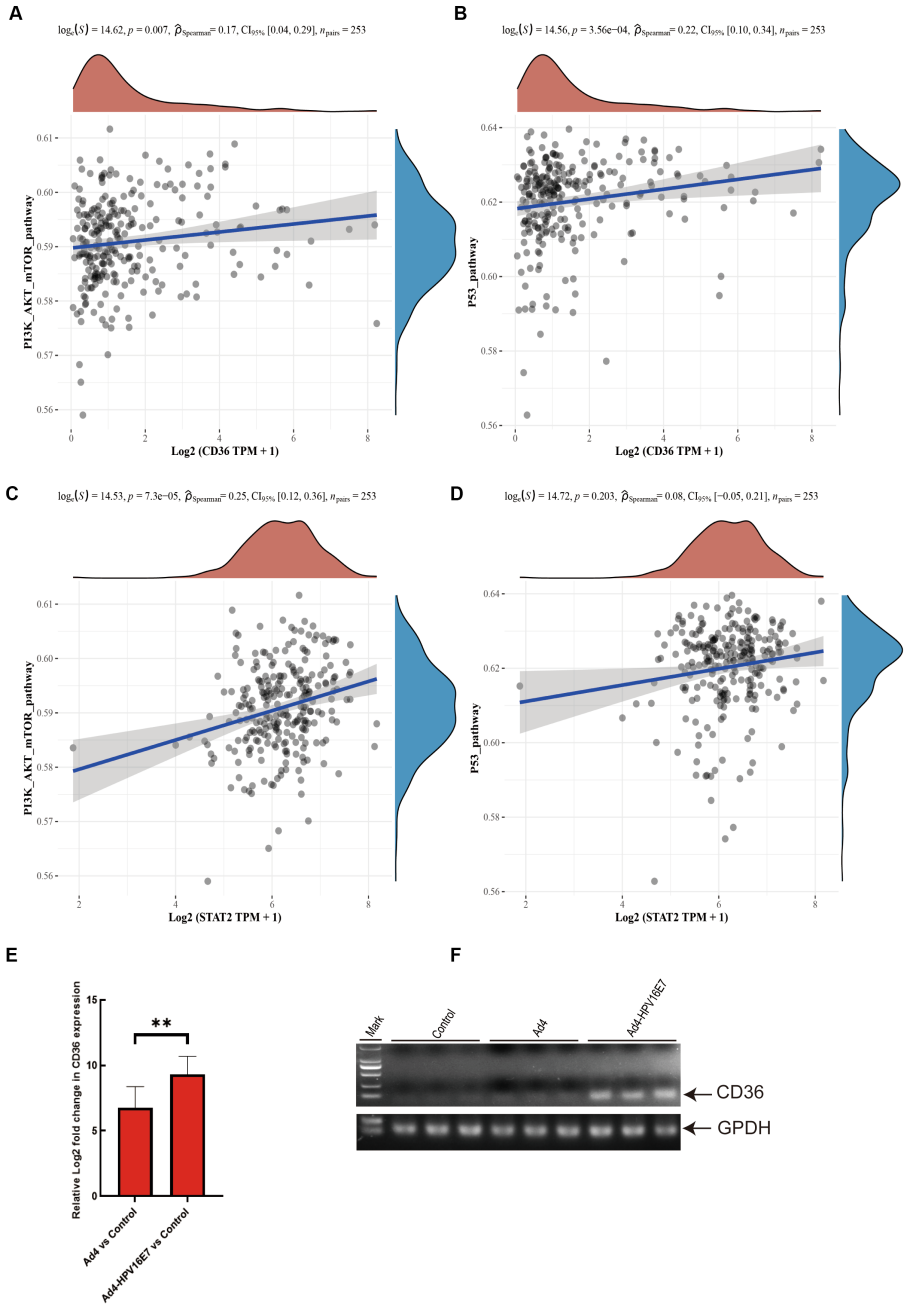
Recombinant virus Ad4-HPV16E7 induces upregulation of CD36 gene in C33A cells. **(A,B)** The Spearman correlation analysis was employed to examine the correlations between individual CD36 and PI3K/AKT/mTOR signaling pathway, as well as the P53 pathway score. **(C,D)** The Spearman correlation analysis was employed to examine the correlations between individual STAT2 and PI3K/AKT/mTOR signaling pathway, as well as the P53 pathway score. *P* < 0.05 was considered statistically significant. **(E)** Detection of the relative expression levels of CD36 gene by qRT-PCR. **(F)** Agarose gel electrophoresis of qRT-PCR amplification products. *P < 0.05 and **P < 0.01 were considered statistically significant.

Moreover, previous studies have provided evidence that the HPV E7 involves in the regulation of the p53 pathway. Activation of the tumor suppressor p53 can induce cell cycle arrest, and the downregulation of cell cycle genes through transcriptional mechanisms is recognized as a primary mechanism in p53-mediated arrest (Nair et al., 2003; Bae et al., 2017; Engeland, 2018). Therefore, we assembled a set of genes associated with cervical cancer-related pathways and employed the ssGSEA algorithm to calculate the scores of the five upregulated genes in the PI3K/AKT/mTOR signaling and p53 pathways. Spearman correlation analysis revealed a statistically significant correlation between the CD36 gene and both the PI3K/AKT/mTOR signaling pathway and the p53 pathway (Figures 8A,B). However, STAT2 exhibited a significant correlation with the PI3K/AKT/mTOR signaling pathway but not with the p53 pathway (Figures 8C,D). There was no statistically significant correlation observed between IFIT1, VMP1, MUC1, and these two pathways.

To further investigate the regulatory impact of the Ad4-HPV16E7 virus on STAT2 and CD36 in HPV-negative cervical cancer cells, C33A cells were infected with Ad4 and Ad4-HPV16E7 viruses, and non-infected C33A cells served as the control group. RNA was extracted from the cells, reverse transcribed into cDNA, and specific primers targeting STAT2 and CD36 were used for qRT-PCR. The results showed that the expression of the CD36 gene was upregulated in the Ad4-HPV16E7 group compared to the Ad4 and control groups. However, there was no significant difference observed in the expression of the STAT2 gene (Figure 8E). Gel electrophoresis of the qRT-PCR products of CD36 confirmed the findings (Figure 8F). These results suggest that the Ad4-HPV16E7 virus can upregulate the expression of the CD36 gene in C33A cells.

## 4. Discussion

The sustained high-level expression of oncogenic proteins E6/E7 is a crucial factor in the development of cancer caused by high-risk HPV infections (Schwartz and Rotter, 1998). The presence of E7 protein is necessary for the malignant transformation of infected tissues, and in many cases of HPV16-associated cancers, the viral genome is integrated into the host genome (Sun et al., 2021). Consequently, these infected cells predominantly express E6/E7 oncogenes, rendering HPV vaccines targeting the L1 and L2 capsid proteins ineffective against established infections (Li et al., 2016; Taghinezhad et al., 2021). Given these unique characteristics of high-risk HPV in carcinogenesis, the E7 proteins emerge as ideal therapeutic targets for HPV-related cancers and represent a focal point for gene therapy research aimed at specifically targeting cells expressing the E7 antigen (He et al., 2019). Targeting E7 not only elicits a specific anti-tumor response in cancer cells but also minimizes the risk of unintended cytotoxicity toward healthy cells. However, the intricate life cycle of HPV and the challenges associated with *in vitro* culturing of the virus have impeded further advancements in related research. To address this limitation, we propose the construction of recombinant viral particles using adenoviral vectors, which provide a stable platform for expressing the HPV16 E7 protein. This approach provides a novel avenue for investigating HPV-associated anti-tumor and antiviral therapies.

The HPV E7 region exhibits homology with Ad E1A, which is known to lack carcinogenic properties in humans. Thus, by inserting the HPV16 E7 gene into the EGFP-tagged Ad4 E1A region, we successfully constructed a replication-competent recombinant virus, Ad4-HPV16E7, for the first time. This was achieved through a combination of the ccdB-Kan positive/negative selection system and the Red/ExoCET recombination system. We infected HEK293T, Siha, caski, Hela, and C33A cells with the recombinant virus and observed that while its infectivity was relatively weak in Hela cells, it exhibited strong infectivity in HEK293T, Siha, caski, and C33A cells. In HEK293T and C33A cells infected with the recombinant virus, HPV16 E7 protein was stably and effectively expressed. Additionally, the expression level of the Hexon protein in the constructed recombinant virus, Ad4-HPV16E7, was higher compared to that of the Ad4 virus, indicating a stronger replication capacity of the recombinant virus relative to Ad4. However, further studies are required to confirm the replication capacity of the recombinant virus.

We performed RNA-Seq analysis to construct libraries and conduct biological analysis of all mRNA transcripts generated by C33A cells after infection with Ad4-HPV16E7 virus. The RNA-Seq results were cross-referenced with reliable sources such as OMIM, Genotype, KEGG, and PubMed to identify genes associated with HPV infection, resulting in the identification of 103 differentially expressed genes (DEGs). Protein–protein interaction (PPI) analysis using STRING network revealed a high level of functional relationship among these 103 DEGs. GO, KEGG, DO, and DisGeNET analyses determined several enriched biological processes and metabolic pathways for these 103 genes. Notably, KEGG pathway analysis indicated significant enrichment of these genes in the PI3K-Akt signaling pathway, which plays a crucial role in HPV-induced carcinogenesis. The clustering analysis of these 103 DEGs indicated that the expression of five upregulated genes (STAT2, CD36, IFIT1, VMP1, and MUC1) in the Ad4-HPV16E7 group was higher than that in the Ad4 and control groups. The selection of these genes was based on the similarity of their expression patterns in the clustering analysis and their association with HPV infection in previous literature. Previous studies have shown that HPV E7 plays a role in regulating the p53 pathway, and activation of the tumor suppressor factor p53 can lead to cell cycle arrest. Additionally, the main mechanism of p53-mediated arrest is the transcriptional downregulation of multiple cell cycle genes. Therefore, we collected genes related to cervical cancer-associated pathways and calculated the correlation scores between these five upregulated genes and the PI3K/AKT/mTOR signaling pathway and the p53 pathway through Spearman correlation analysis. The results indicated a significant correlation between the CD36 gene and both the PI3K/AKT/mTOR signaling pathway and the p53 pathway. The STAT2 gene showed a significant correlation with the PI3K/AKT/mTOR signaling pathway but not with the p53 pathway. IFIT1, VMP1, and MUC1 showed no significant correlation with either of these pathways. qRT-PCR results demonstrated upregulation of the CD36 gene expression in the Ad4-HPV16E7 group compared to the Ad4 and control groups. These findings suggest that the Ad4-HPV16E7 virus has the ability to upregulate CD36 gene expression in C33A cells. Alterations in CD36 expression may be associated with high-risk human papillomavirus infection and may promote the development and progression of cervical cancer. Several studies have shown that CD36 is involved in cell proliferation by regulating the cell cycle. The pathway analysis revealed the correlation between CD36 and the PI3K/AKT/mTOR signaling pathway, which is consistent with previous research and further strengthens the potential role of CD36 in HPV infection and cervical cancer. These results provide important clues for further research into the molecular mechanisms underlying HPV infection and cervical cancer development.

This study investigates a cost-effective and precise approach to constructing a replicative recombinant virus that expresses the HPV16 E7 protein and the successful expression of HPV16 E7 in cells demonstrated that the replicated recombinant virus maintains the replication and infection capabilities of Ad4, while also upregulated the CD36 gene, which is involved in the PI3K-Akt signaling and p53 pathways for promoting cell proliferation. The findings of this study offer a novel perspective for future investigations on HPV-related carcinogenesis/disease and the advancement of replicative recombinant HPV therapeutic vaccines that can elicit protective immunity against HPV. Absolutely, the use of recombinant adenovirus for gene therapy suffers from some challenges. The recombinant adenovirus possesses the tropism of the parental viruses, which infect all cells that possess the appropriate surface receptors, precluding the targeting of specific cell types. Conversely, some cell types that represent important tar gets for gene transfer express only low levels of the cellular receptors, which lead to inefficient infection. Thus, there is a need for addressing attention the rational strategies based on the biology of Ad to further exploit the full potential of the recombinant adenovirus for *in vivo* gene delivery upon systemic administration, thereby provide the basis for researching the replicative recombinant HPV therapeutic vaccines.

## Data availability statement

The data presented in the study are deposited in the NCBI Gene Expression Omnibus (GEO, http://www.ncbi.nlm.nih.gov/geo), accession number GSE240750.

## Ethics statement

Ethical approval was not required for the studies on humans in accordance with the local legislation and institutional requirements because only commercially available established cell lines were used.

## Author contributions

YS: Data curation, Investigation, Software, Writing – original draft. YZ: Investigation, Writing – review & editing. HC: Software, Writing – original draft. ZL: Software, Writing – review & editing. PW: Methodology, Resources, Writing – review & editing. CW: Conceptualization, Funding acquisition, Project administration, Resources, Writing – review & editing. SL: Participated in the data analysis, Edited the manuscript. FH: Conducted the experiments, Participated in the data analysis.

## Funding

The author(s) declare financial support was received for the research, authorship, and/or publication of this article.

This study was supported by grants from the National Natural Science Foundation of China (No. 82072287) and National Key Research and Development Program of China [2018YFA0900800].

## Conflict of interest

The authors declare that the research was conducted in the absence of any commercial or financial relationships that could be construed as a potential conflict of interest.

## Publisher’s note

All claims expressed in this article are solely those of the authors and do not necessarily represent those of their affiliated organizations, or those of the publisher, the editors and the reviewers. Any product that may be evaluated in this article, or claim that may be made by its manufacturer, is not guaranteed or endorsed by the publisher.

## Footnotes

^1^ http://www.rcsb.org/pdb/home/home.do

^2^ http://autodock.scripps.edu/

^3^ http://rsbweb.nih.gov/ij/

^4^ http://www.genome.jp/kegg/

^5^ http://string-db.org

^6^ https://www.ncbi.nlm.nih.gov/omim

^7^ https://www.ncbi.nlm.nih.gov/gap/phegeni

^8^ https://pubmed.ncbi.nlm.nih.gov
