# Supplementary material for: A replicative recombinant HPV16 E7 expression virus upregulates CD36 in C33A cells
Source: Front Microbiol. 2023 Aug 30;14:1259510. doi: 10.3389/fmicb.2023.1259510 (PMC10545859; doi:10.3389/fmicb.2023.1259510)
Supplement: Supplementary file 1 [file Data_Sheet_1.PDF]

Supplementary Material

Supplementary Table1

All *Escherichia coli* strains and plasmids evaluated in this study.

Strains/ Reference/Source	Genotype/Description	Reference/Source
Strains		
E. coli DH5α	F- φ80 *lac* ZΔM15 Δ(*lac*ZYA-*arg* F) U169 *end*A1 *rec*A1 *hsd*R17(rk-,mk+) *sup*E44λ- *thi* -1 *gyr*A96 *rel*A1 *pho*A	Invitrogen (CA, USA)
E. coli DH10B	F- *mcr*A Δ(*mrr*-*hsd*RMS-*mcr*BC) φ80*lac*ZΔM15 Δ*lac*X74 *rec*A1 *end*A1 *ara*D139 Δ(*ara*, *leu*)7697 *gal*E15 *gal*K λ- *rps*L *nup*G	Invitrogen (CA, USA)
GB08-Red	*E. coli* strain harboring an arabinose inducible γβαA operon (redγ, redβ, redα and recA) at the ybcC locus	(Fu, Teucher et al. 2010)
GB05-dir	derived by integrating the PBAD-ETgA operon into the ybcC locus in GB2005	(Fu, Bian et al. 2012)
Plasmids		
Ad4	GenBank accession no. AY594253	(Lin, Wang et al. 2006)
pR6K-kan-ccdB	pR6K origin, Kan^R	This study
HPV16	NCBI Reference Sequence: NC_001526.4	(Liu, Shen et al. 2016)

Reference

Fu, J., X. Bian, S. Hu, H. Wang, F. Huang, P. M. Seibert, A. Plaza, L. Xia, R. Müller, A. F. Stewart and Y. Zhang (2012). "Full-length RecE enhances linear-linear homologous recombination and facilitates direct cloning for bioprospecting." Nat Biotechnol **30**(5): 440-446.

Fu, J., M. Teucher, K. Anastassiadis, W. Skarnes and A. F. Stewart (2010). "A recombineering pipeline to make conditional targeting constructs." Methods Enzymol **477**: 125-144.

Lin, B., Z. Wang, G. J. Vora, J. A. Thornton, J. M. Schnur, D. C. Thach, K. M. Blaney, A. G. Ligler, A. P. Malanoski, J. Santiago, E. A. Walter, B. K. Agan, D. Metzgar, D. Seto, L. T. Daum, R. Kruzelock, R. K. Rowley, E. H. Hanson, C. Tibbetts and D. A. Stenger (2006). "Broad-spectrum respiratory tract pathogen identification using resequencing DNA microarrays." Genome Res **16**(4): 527-535.

Liu, Q., Q. Shen, X. Bian, H. Chen, J. Fu, H. Wang, P. Lei, Z. Guo, W. Chen, D. Li and Y. Zhang (2016). "Simple and rapid direct cloning and heterologous expression of natural product biosynthetic gene cluster in Bacillus subtilis via Red/ET recombineering." Sci Rep **6**: 34623.
